# Microbial superoxide production drives biogenic nitrogen dioxide formation in soils

**DOI:** 10.1038/s41467-026-76881-x

**Published:** 2026-08-17

**Authors:** Megan L. Purchase, Jonathan D. Raff, Deying Wang, Ryan M. Mushinski

**Affiliations:** 1 School of Life Sciences, University of Warwick, Coventry, UK; 2 Paul H. O’Neill School of Public and Environmental Affairs, Indiana University, Bloomington, IN USA

**Keywords:** Microbial ecology, Element cycles

## Abstract

Nitrogen dioxide (NO_2_) is a critical atmospheric pollutant and ozone precursor, yet biogenic soil sources remain poorly constrained. Current models assume soil NO_2_ flux is exclusively depositional. Here we demonstrate that soils can produce NO_2_ through microbial superoxide (O_2_ˉ) production. Using controlled factorial slurry experiments, native microbial communities produced approximately 10 times more NO_2_ than sterile controls following nitric oxide (NO) exposure. Stimulating superoxide production with NADH increased NO_2_ formation 15- to 26-fold, while inhibiting NADH oxidase reduced production toward baseline levels. Superoxide dismutase decreased NO_2_ production by 46-71%, and O_2_ˉ concentration explained 60% of variation in NO_2_ production rates. Addition of peroxynitrite to soil increased headspace NO_2_, confirming this intermediate as the mechanistic link. These findings reveal a pathway linking carbon and nitrogen cycling where heterotrophic decomposers facilitate biogenic NO-to-NO_2_ conversion via superoxide chemistry, potentially explaining discrepancies between satellite observations and modelled soil NO_x_ emissions.

## Abstract (web summary)

Soil microbes produce superoxide, which reacts with nitric oxide to form nitrogen dioxide directly in soil. This overlooked pathway means soils can emit NO₂ as well as NO, with implications for how nitrogen emissions are modelled.

## Introduction

Soils contribute approximately 15% of the global land-to-atmosphere NO_x_ flux, estimated at 9–10 Tg N y^−1^^1^. Current biogeochemical models treat these emissions as exclusively nitric oxide (NO), assuming any NO_2_ in the soil-atmosphere exchange results entirely from atmospheric oxidation of emitted NO or from deposition. However, satellite observations consistently reveal discrepancies with bottom-up model estimates. Pre-2012 models underestimated observed NO_2_ columns by factors of 2–4 over North America and Africa, and recent studies suggest natural NO_x_ from Amazonian and agricultural soils may be 10–20 times larger than current model inventories^2–4^. While empirical parameterisation schemes have improved considerably over recent decades, process-level understanding of soil NO_x_ speciation, particularly the conditions governing NO_2_ versus NO emission, remains limited. These persistent discrepancies suggest undefined pathways for gaseous NO_x_ production from soil, potentially including direct biogenic NO_2_ formation.

Recent advances in understanding microbial reactive oxygen species production offer a potential mechanism for biogenic NO_2_ formation. Heterotrophic bacteria and fungi express extracellular and cell-surface NAD(P)H-dependent oxidoreductases, including NADPH oxidase (NOX)-family flavoenzymes, that reduce O_2_ to superoxide (O_2_ˉ)^5^. In physiological systems, O_2_ˉ reacts rapidly with NO at near diffusion-limited rates (*k* > 10^9 ^M^−1^ s^−1^) to form peroxynitrite (ONOO^–^)^6^, which subsequently decomposes to yield NO_2_ and other reactive nitrogen species^7^. We hypothesised that this well-characterised physiological chemistry could occur in soils where nitrogen-cycling microorganisms produce NO through nitrification and denitrification while heterotrophic decomposers produce superoxide.

## Results

**Fig. 1:**
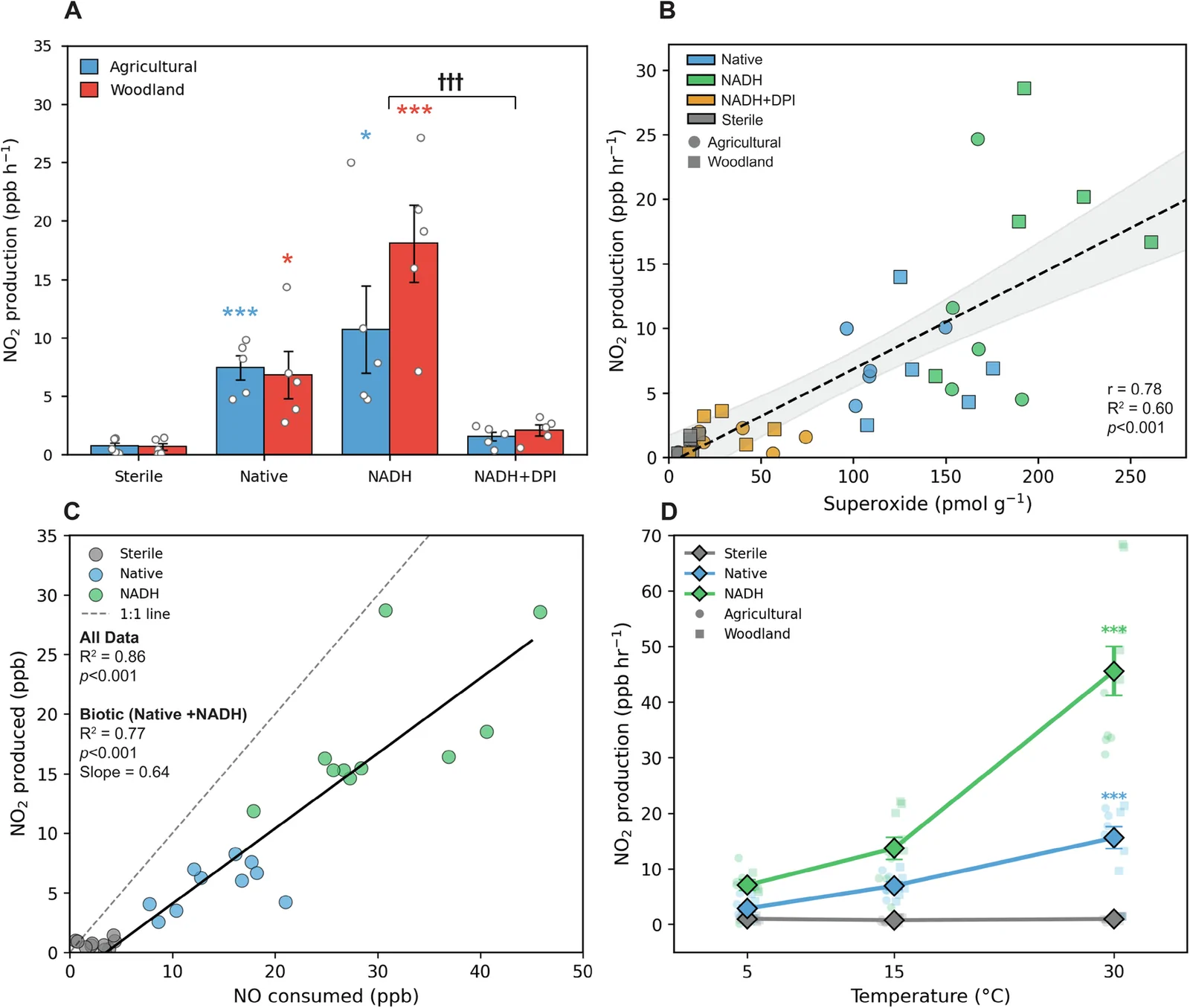
Biogenic NO_2_ production from soil microbial activity. **A** NO_2_ production rates across treatments in agricultural (blue) and woodland (red) soils. Data are presented as mean values ± S.E.M. with individual replicates overlaid. *n* = 5 biologically independent soil incubations per treatment per soil; the unit of study is a single incubation vessel derived from one of five biological soil samples per site. Treatments (sterile, native, NADH, NADH + DPI) are compared against the sterile control. Treatment means were compared with the sterile control by two-sided two-sample t-test (per soil); NADH was compared with NADH + DPI by two-sided two-sample t-test with data pooled across both soils. *p < 0.05, ***p < 0.001 vs. sterile; ^†††^*p* = 0.001, NADH vs. NADH + DPI. Exact P values are given in Source Data. **B** Correlation between soil superoxide concentration and NO_2_ production rate across all treatments (Pearson’s r = 0.78, R^2^ = 0.60, two-sided *p* < 0.001; n = 40 biologically independent incubations, 5 per treatment per soil). The dashed line is the ordinary least-squares fit and the shaded band the 95% confidence interval. Circles, agricultural; squares, woodland. **C** Relationship between NO consumed and NO_2_ produced. Points are individual biologically independent incubations (*n* = 30; sterile, native and NADH, 5 per treatment per soil). The solid line is the linear fit to the biotic treatments (native + NADH; slope = 0.64, indicating 64% conversion efficiency; R^2^ = 0.77, two-sided *p* < 0.001); the dashed grey line is the 1:1 relationship. Fits are ordinary least-squares linear regression. **D** Temperature dependence of NO_2_ production. Data are presented as mean values ± S.E.M. with individual replicates overlaid; *n* = 10 biologically independent incubations per treatment per temperature (5 per soil, pooled across agricultural and woodland soils), with the incubation vessel as the unit of study. Sterile, native and NADH treatments are shown; ***p < 0.001 vs. 5 °C (two-way ANOVA with temperature and treatment as factors, two-sided; exact P values in Source Data).

Using controlled factorial slurry experiments with agricultural (sandy loam, pH 6.8, 3.2% organic matter) and woodland (clay loam, pH 5.9, 8.1% organic matter) soils from Warwickshire, UK, we tested this mechanism through selective stimulation and inhibition of superoxide-producing pathways. Sterile soil showed minimal NO_2_ accumulation following NO addition (agricultural: 0.72 ± 0.62 ppb h^−1^; woodland: 0.68 ± 0.66 ppb h^−1^), representing baseline abiotic conversion. In contrast, native soils with intact microbial communities produced significantly elevated NO_2_, roughly 10-fold higher than sterile controls in both soils (agricultural: 7.46 ± 2.31 ppb h^−1^, *p* < 0.001; woodland: 6.84 ± 4.54 ppb h^−1^, *p* < 0.05; Fig. 1A). This demonstrates that soil microorganisms actively convert NO to NO_2_ under aerobic conditions. This superoxide-driven pathway is inherently aerobic, as extracellular O_2_¯ production by heterotrophic NADH oxidases requires molecular oxygen as the terminal electron acceptor. The mechanism is therefore expected to be most significant in surface and near-surface soil horizons where aerobic conditions prevail, and biogenic NO_x_ emissions occur.

NADH addition to stimulate extracellular superoxide production via NADH oxidase activity increased NO_2_ formation, with 15-fold increase relative to sterile controls in agricultural soil (10.71 ± 8.35 ppb h^−1^; *p* < 0.05) and 26-fold increase in woodland soil (18.07 ± 7.35 ppb h^−1^; *p* < 0.001). When diphenyleneiodonium (DPI) was co-applied to inhibit flavin-containing NADH oxidases, NO_2_ production fell sharply relative to NADH-stimulated slurries (NADH vs NADH + DPI, *p* = 0.001), approaching baseline, indicating that NADH oxidase activity is necessary for biogenic NO_2_ production. Concurrent measurement of extracellular superoxide revealed a strong positive correlation with NO_2_ production across all treatments and soil types (r = 0.78, R^2^ = 0.60, *p* < 0.001; Fig. 1B), supporting the proposed mechanism. The conversion efficiency of NO-to-NO_2_ was approximately 64% in biotic treatments (Fig. 1C), and production rates increased strongly with temperature (Fig. 1D), consistent with enzymatic processes.

**Fig. 2:**
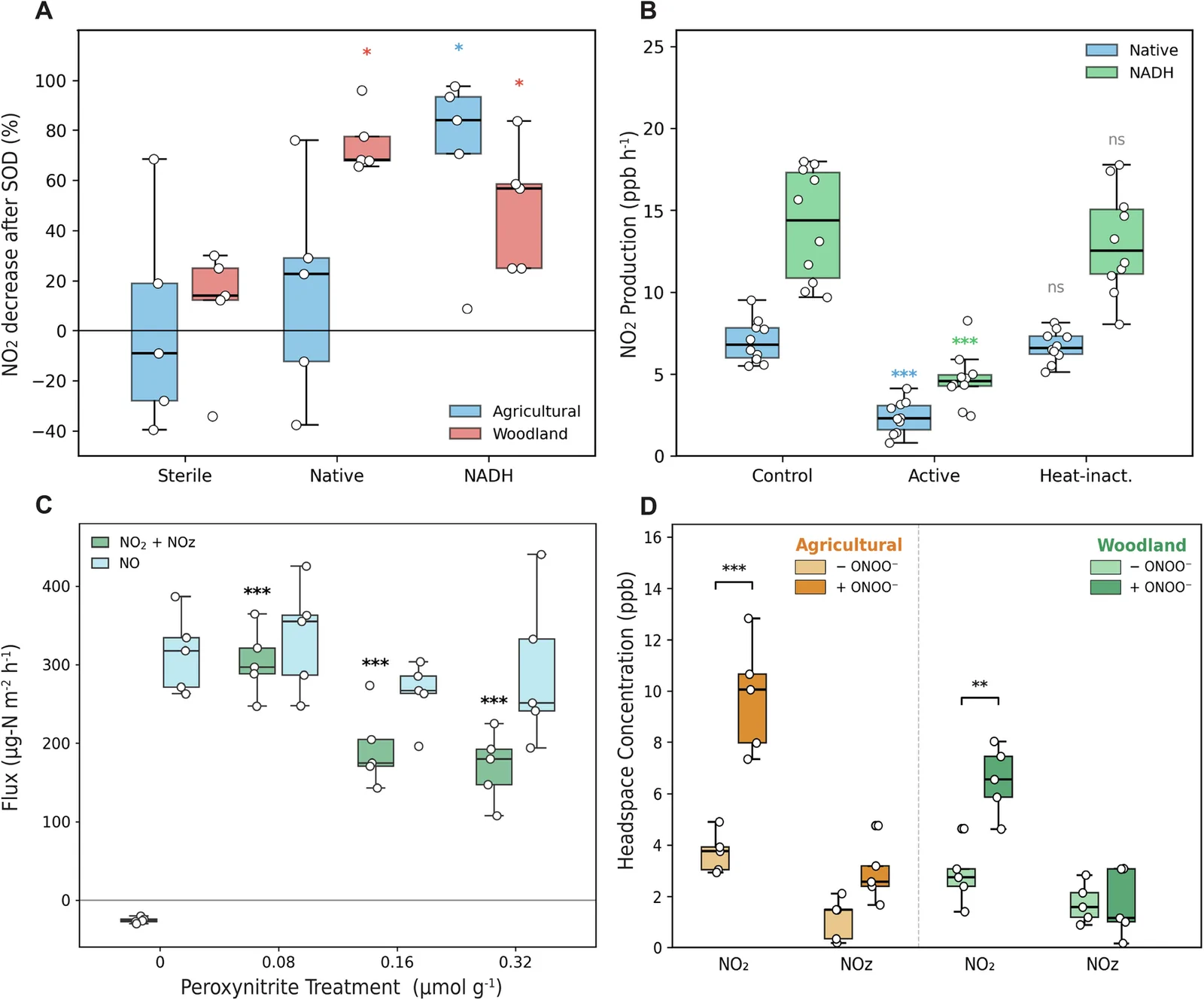
Superoxide and peroxynitrite mediate NO_2_ formation. A Percent decrease in NO_2_ production rate with superoxide dismutase (SOD; 500 U ml^−1^) relative to sterile, native and NADH-stimulated slurries in agricultural (blue) and woodland (red) soils. *n* = 5 biologically independent soil incubations per treatment per soil. Positive values indicate SOD-sensitive, superoxide-dependent NO_2_ production. *p < 0.05 for a reduction significantly greater than zero (paired *t-*test, two-sided; exact *P* values in Source Data). B NO_2_ production rate in native and NADH-stimulated treatments with no SOD (control), active SOD, or heat-inactivated SOD (100 °C, 10 min). *n* = 10 biologically independent incubations per box (5 per soil, pooled across agricultural and woodland soils); the unit of study is a single incubation vessel. Heat inactivation abolishes enzymatic activity, confirming that the active-SOD effect reflects superoxide scavenging rather than non-specific protein effects. ***p < 0.001; ns, not significant (active and heat-inactivated SOD compared to the no-SOD control by two-sample *t-*test, two-sided; exact P values in Source Data). **C** NO and NO_2_ + NO_z_ flux in response to peroxynitrite (ONOO⁻) applied to sterile soil microcosms at 0, 0.08, 0.16 and 0.32 µmol g^−1^. *n *= 5 biologically independent microcosms per dose per gas species; the unit of study is a single microcosm. The negative flux at 0 µmol g^−1^ reflects NO_2_ deposition to sterile soil in the absence of peroxynitrite and serves as the baseline control. ***p < 0.001 vs. the 0 µmol g^−1^ baseline (one-way ANOVA with Tukey’s HSD, two-sided; exact P values in Source Data). **D** Headspace NO_2_ and NO_z_ concentrations in agricultural and woodland soil slurries following addition of synthesised peroxynitrite (+ONOO¯) or vehicle (−ONOO¯). *n* = 5 biologically independent slurries per condition; the unit of study is a single slurry vessel. ***p < 0.001, **p < 0.01 ( + ONOO¯ vs. −ONOO¯, unpaired *t-*test, two-sided; exact P values in Source Data). In all box plots the centre line is the median, box bounds are the 25th and 75th percentiles, and whiskers extend to the most extreme points within 1.5 x the interquartile range; all individual data points are overlaid.

To directly test whether superoxide is required for NO_2_ formation rather than simply correlated, we added superoxide dismutase (SOD) to convert O_2_ˉ to hydrogen peroxide (H_2_O_2_) before it could react with NO. SOD was applied to a separate vessel prepared from each of the same five biological soil samples used for the untreated (no-SOD) slurries in Fig. 1A, so that SOD-treated and untreated rates are paired within biological samples. In woodland soil, active SOD reduced NO_2_ production by 71% in native treatments (6.84 ± 4.54 to 1.98 ± 1.70 ppb h^−1^; paired *t-*test, *p* < 0.05) and by 46% in NADH treatments (18.07 ± 7.35 to 9.71 ± 5.52 ppb h^−1^; *p* < 0.05; Fig. 2A). Importantly, heat-inactivated SOD (boiled 10 min) had no significant effect on NO_2_ production (*p* > 0.05; Fig. 2B), confirming that the observed reduction is due to enzymatic superoxide scavenging rather than non-specific protein effects.

**Fig. 3:**
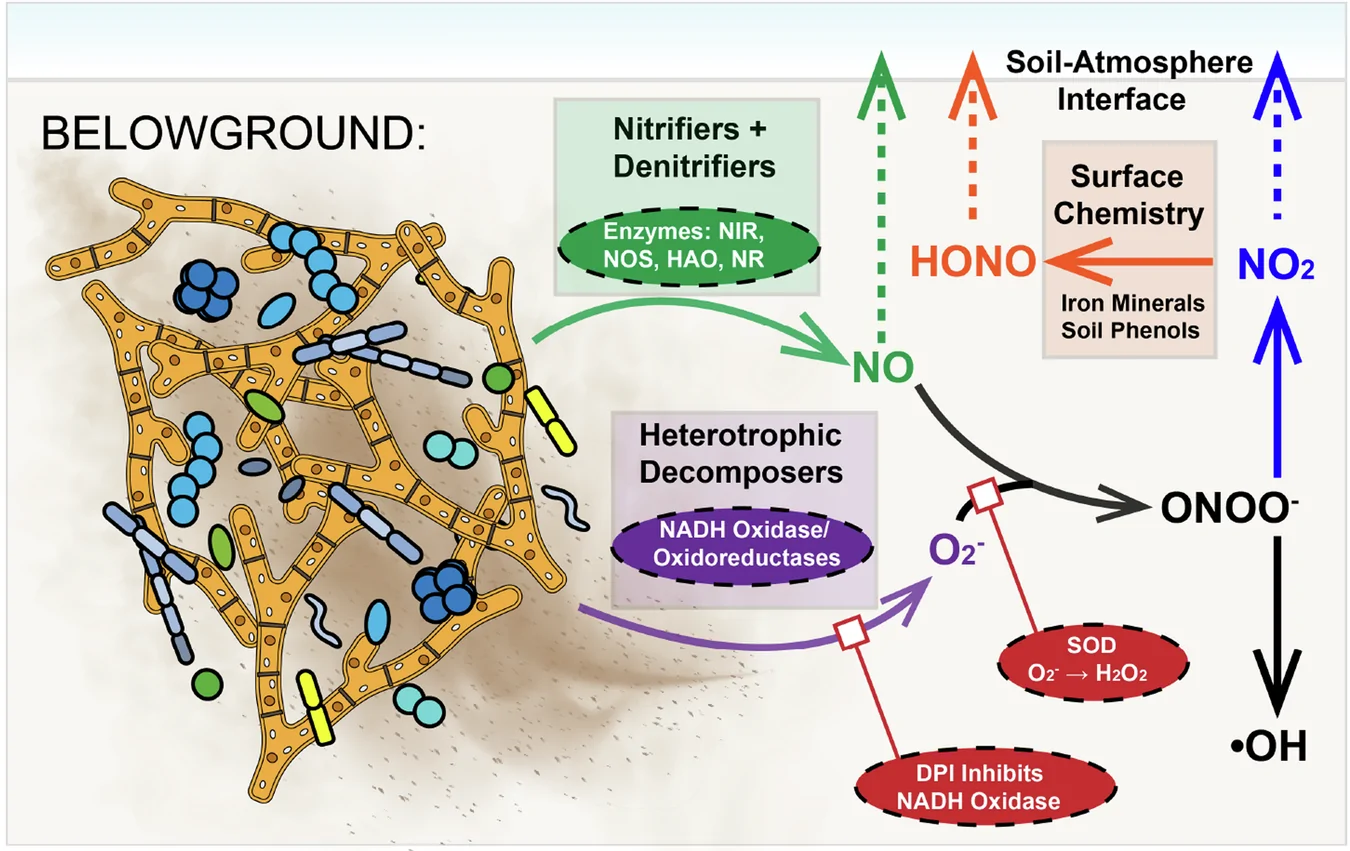
Proposed mechanism for biogenic NO_2_ production from soils. Nitrifiers and denitrifiers produce nitric oxide (NO) via key nitrogen-cycling enzymes (NIR, NOS, HAO, NR). Heterotrophic decomposers generate extracellular superoxide (O_2_ˉ) through NADH oxidase and oxidoreductases. NO and O_2_ˉ react to form peroxynitrite (ONOO¯), which decomposes to NO_2_ and hydroxyl radical (•OH). NO_2_ is emitted across the soil-atmosphere interface or converted to HONO_(g)_ via surface chemistry involving iron minerals and soil phenols. Red inhibitors show experimental controls of the superoxide mechanism, where diphenyleneiodonium (DPI) blocks NADH oxidase; superoxide dismutase (SOD) scavenges O_2_ˉ, confirming superoxide-dependent NO_2_ formation. A dashed green arrow indicates direct NO emission from soil to the soil-atmosphere interface, consistent with the well-established role of soils as net NO sources under most biogeochemical conditions.

If the proposed mechanism is correct, that superoxide reacts with NO to form peroxynitrite, which then decomposes to NO_2_, then direct addition of peroxynitrite to soil should enhance NO_2_ emissions. Addition of synthesised ONOO^–^ (0.08-0.32 µmol g^-1^) to soil microcosms significantly increased NO_2_ + NO_z_ [NO_z_ = NO_y_ - NO_x_] fluxes compared to water controls (*p* < 0.001 for all concentrations vs. baseline; Fig. 2C). Additional soil validation experiments confirmed that peroxynitrite addition significantly increased NO_2_ concentrations across agricultural and woodland soils (Fig. 2D). This confirms that peroxynitrite decomposition in soil produces NO_2_ that can be emitted past the soil-atmosphere interface. All experimental components combined, as depicted in Fig. 3, the proposed mechanism proceeds through the reaction, NO + O_2_ˉ → ONOO^–^, followed by peroxynitrite decomposition to yield NO_2_ and hydroxyl radical ^8^.

## Discussion

The approximately 29-54% residual NO_2_ production following SOD addition likely reflects the operation of multiple parallel peroxynitrite decomposition pathways that are not intercepted by superoxide scavenging. While SOD prevents ONOO¯ formation by converting O_2_¯ to H_2_O_2_ before it can react with NO, peroxynitrite chemistry in soil is not limited to a single fate. Hydroperoxyl radical, HO_2_•, the protonated form of O_2_¯, which predominates below pH 4.8 and is poorly scavenged by SOD^9^, would represent a minor fraction under our bulk soil conditions, though local pH microenvironments in the soil matrix cannot be excluded. At our bulk soil pH values of 5.9 and 6.8, peroxynitrous acid (ONOOH; pKa 6.8) predominates or co-exists with the anion in approximately equal proportions (88:12 and 50:50 ONOOH:ONOO¯, respectively). Although the major terminal product of ONOOH decomposition is nitrate, NO_2_ and hydroxyl radical (•OH) are obligate intermediates, with nitrate formed only when NO_2_ and •OH recombine within the solvent cage^7^. In soil, abundant organic matter and bicarbonate function as competitive •OH scavengers, kinetically hindering cage recombination and increasing the probability that NO_2_ escapes into the gas phase before reacting with •OH. Additionally, ONOO¯ reacts with dissolved CO_2_ to form the adduct ONOOCO_2_¯, which decomposes to yield NO_2_ and carbonate radical anion (CO_3_•¯) in approximately 30–40% yield^10,11^. Given that soil CO_2_ concentrations substantially exceed those of dilute aqueous systems due to active respiration, this pathway likely operates toward the higher end of this yield range in soil, representing an additional source of NO_2_ that operates independently of bulk pH speciation. Additionally, Fe and Mn redox-active minerals abundant in soil have been shown to decompose peroxynitrite via one-electron oxidation pathways that generate NO_2_^12,13^, consistent with the established role of iron minerals in soil reactive nitrogen chemistry^14,15^. Together, these pathways explain why NO_2_ production is inhibited but not abolished by SOD addition, and why the mechanism is expected to remain operative across the range of pH conditions encountered in natural soils.

The soil physicochemical conditions most likely to favour this mechanism can be identified from first principles. High organic matter content supports the diverse heterotrophic communities responsible for extracellular superoxide production^5^, consistent with the high superoxide production potential of the woodland soil. The similar absolute NO_2_ production rates observed across the two contrasting soil types (7.46 ± 2.31 and 6.84 ± 4.54 ppb h^-1^ in agricultural and woodland soils, respectively) support the co-dependence of the mechanism on both NO substrate availability and superoxide production capacity. The higher NO_3_¯ availability in the agricultural soil (Table S1) is consistent with greater nitrification activity and NO substrate supply, while the higher NH_4_⁺ and organic matter content of the woodland soil supports greater heterotrophic superoxide production capacity. That both soils converge on similar NO_2_ production rates despite these contrasting properties suggests the mechanism is robust across a range of soil biogeochemical conditions where the two requirements are met through different combinations of soil properties. Circumneutral to slightly acidic pH (approximately 5.5–7.5) favours both nitrification^16^ and denitrification^17^, ensuring the NO substrate supply necessary for the mechanism, while also maintaining the ONOOH:ONOO¯ speciation that enables parallel decomposition pathways described above. Adequate aeration is a prerequisite given the aerobic nature of the superoxide-producing enzymes involved. In terms of land-use scenarios, the mechanism is therefore likely to be most significant in three contexts, fertilised agricultural soils following nitrogen application, where simultaneous stimulation of nitrifier activity and heterotrophic decomposition of organic amendments creates the necessary co-occurrence of NO and superoxide production^5,18^; organic-rich forest soils where high heterotrophic activity is sustained year-round and nitrogen cycling supports background NO production^19^; and soils experiencing wet-dry transition events, where rewetting pulses transiently stimulate both microbial communities simultaneously^18^. Conversely, highly acidic soils (pH <5), waterlogged anaerobic conditions, or soils with very low organic matter content would be expected to suppress the mechanism through inhibition of nitrification, elimination of aerobic superoxide production, or insufficient heterotrophic biomass, respectively.

These findings have important implications for understanding previous measurements of soil NO_x_ budgets and the persistent discrepancies between satellite observations and bottom-up models. Current biogeochemical models do not include this pathway, potentially leading to systematic underestimation of soil NO_x_ emissions. Our results extend previous observations that some forest soils emit NO_2_ rather than acting exclusively as sinks^19^, providing a mechanistic framework explaining why emission versus deposition depends on soil biogeochemical conditions. Soils with high organic matter, supporting heterotrophic superoxide production, combined with active nitrogen cycling would favour NO_2_ production and potential emission. Soil emissions of NO_2_ have been previously reported; for example, Williams et al., using chambers in the field estimated that NO_2_ fluxes were 10% those of NO^20^. However, the origin of the emissions was not clear at the time. More recently, Shah et al. confirmed positive NO_2_ fluxes from agricultural soils under realistic moisture conditions using a validated dynamic chamber system, while noting that the scarcity of such measurements reflects methodological limitations rather than the absence of the process^21^. Our work also implies that some of the HONO_(g)_ soil emissions reported could stem from NO_2_-to-HONO conversion on soil surfaces as previously described^14,15^. Thus, it is possible that NO_2_ formation in soil has been underreported due to its rapid conversion into HONO on soil surfaces. Further, recent updates to NO_x_ dry deposition parameterisations confirm that NO_2_ heterogeneous hydrolysis on soil and canopy surfaces is substantially underrepresented in current models, with nocturnal deposition velocities underestimated by approximately 80%^22^. Canopy reduction factors of 47–59% calculated for forest ecosystems suggest that a substantial fraction of soil-emitted NO is intercepted before reaching above-canopy measurement heights^22^; biogenic NO_2_ would be intercepted even more efficiently given its greater surface reactivity. Furthermore, direct measurements of soil pore-space NO concentrations in fertilised agricultural systems have reported average growing-season values of up to 3300 ppb following N fertilisation^23^, with surface ambient NO concentrations of 170 ppb recorded over fertilised soils representing a substantial underestimate of subsurface pore-space concentrations due to diffusion and dilution^24^, demonstrating that soil pore-space NO concentrations under field conditions can substantially exceed the 45 ppb used in our experiments, making our experimental design conservative rather than extreme.

 By showing that heterotrophic activity directly influences reactive nitrogen emissions, these findings reveal a link between terrestrial carbon and nitrogen cycling. Current process-based biogeochemical models that simulate soil NO_x_ emissions, from plot-scale frameworks to the land surface components of Earth system models including CLM5 and ELM, treat NO production as the exclusive output of nitrification and denitrification, with NO_2_:NO ratios at the soil-atmosphere interface determined by assumed atmospheric chemistry rather than soil biological activity^2,25,26^. As a result, modelled NO_2_:NO ratios are insensitive to organic matter availability and heterotrophic community activity, meaning that management practices known to alter decomposer biomass and activity, including tillage, organic matter amendments, and cover cropping, have no representation in current model frameworks with respect to reactive nitrogen gas speciation. Incorporating the mechanism described here would require representing extracellular superoxide production as a function of heterotrophic respiration rate and organic matter availability, parameters already tracked by most process-based models, making implementation tractable without the addition of new state variables. Furthermore, because NO_2_ produced in soil is subject to rapid surface conversion to HONO via iron mineral and phenol chemistry, Earth system models that currently treat soil-atmosphere HONO exchange as exclusively abiotic may be missing a biologically mediated source term that scales with organic matter decomposition and would therefore respond to land-use change and soil management in ways that purely abiotic parameterisations cannot capture. While direct incorporation into empirically parameterised NO_x_ inventories will require further field-scale quantification, this mechanism warrants representation in process-based Earth system models and may account for a component of the HONO emissions increasingly reported from soil surfaces globally.

## Methods

### Soil collection and characterisation

Soils were collected in September 2024 from two contrasting land-use types at the University of Warwick’s Stratford Innovation Campus (52.205°N, 1.602°W). The agricultural site was under continuous winter wheat cultivation (cv. Skyfall) with conventional tillage and fertilisation practices (120 kg N ha^-1^ y^-1^ as urea). The woodland site was a mature mixed deciduous woodland dominated by *Quercus robur* and *Fraxinus excelsior* with no management intervention for >30 years. At each site, five replicate sampling points were established along a 30 m transect with 5 m spacing. At each point, surface litter was removed and soil cores (5 cm diameter x 15 cm depth) were collected using sterile PVC corers. Cores were immediately placed in sterile polyethylene bags, stored on ice, and transported to the laboratory within 2 h of collection. Soils were homogenised by passing through a 2 mm stainless steel sieve. Sieved soils were stored at 4 °C until use. Soil physicochemical properties were determined using standard methods. Soil texture was analysed using the hydrometer method. Soil pH was measured in a 1:2 (w/v) soil:deionised water suspension using a calibrated glass electrode (Mettler Toledo). Organic matter content was determined by loss-on-ignition at 550 °C for 4 h. Total carbon and nitrogen were quantified by dry combustion (Elementar vario PYRO cube). Gravimetric water content was determined by oven-drying at 100 °C for 24 h, and water holding capacity (WHC) was measured using the funnel method with 24-hour drainage. All soil physicochemical data are shown in Supplementary Table S1.

### Soil sterilisation

Sterile controls were prepared by autoclaving following validated protocols^27^. Fresh sieved soil (100 g) was placed in autoclavable polypropylene containers with loosened lids and autoclaved at 121 °C and 103 kPa (15 psi) for 30 min. Soils were allowed to cool to room temperature, mixed thoroughly, and the process repeated twice more at 24-hour intervals to eliminate heat-resistant endospores that may germinate between cycles. Sterilisation efficacy was verified by absence of CO_2_ evolution over 7 days at 25 °C and no colony formation on tryptic soy agar plates inoculated with soil suspensions. Sterile soils were stored at 4 °C and used within 24 h of the final autoclave cycle.

### Slurry incubation experiments

Aerobic soil slurries were prepared by combining soil (100 g dry weight equivalent) with sterile deionised water (60 ml) in 250 ml borosilicate glass bottles (Wheaton) fitted with butyl rubber septa. The soil:water ratio (1:0.6 w/v) was selected to maintain aerobic conditions while ensuring adequate mixing and reagent diffusion. Aerobic slurries were selected over homogenised dry soils to ensure homogeneous distribution of chemical amendments throughout the soil matrix, which we found essential for the mechanistic inhibition and stimulation experiments central to this study, and to provide a sufficient aqueous phase for superoxide quantification via the hydroethidine and cytochrome c reduction assays. We acknowledge that slurry conditions may enhance diffusion of reactive species relative to intact soil, potentially increasing measured NO_2_ production rates relative to field conditions; however, the primary purpose of these experiments was mechanistic demonstration rather than quantitative flux estimation, and the relative differences between treatments are robust to this consideration.

Slurries were pre-incubated at 25 °C with orbital shaking (150 rpm) for 24 h to equilibrate microbial activity before experimental treatments. Gas diffusion was maintained during pre-incubation with a 23-gauge needle in the butyl stopper. Four experimental treatments were applied (*n* = 5 biological replicates per treatment per soil type): (i) Sterilised soil with no amendments (sterile); (ii) Untreated soil with intact microbial communities (Native); (iii) Native soil amended with reduced β-nicotinamide adenine dinucleotide (NADH; 0.3 µmol g^-1^ soil; Sigma-Aldrich, ≥97% purity); (iv) Native soil amended with NADH (0.3 µmol g^-1^) plus diphenyleneiodonium chloride (NADH + DPI; 10 µM final concentration; Sigma-Aldrich, ≥98% purity). NADH was dissolved in sterile deionised water immediately before use and added to slurries via syringe injection through the septum. DPI was dissolved in dimethyl sulfoxide (DMSO; final DMSO concentration <0.1% v/v) and pre-incubated with slurries for 30 min before NADH addition to allow enzyme inhibition. Control treatments received equivalent volumes of vehicle (water or DMSO). The NADH concentration (0.3 µmol g^-1^) was selected based on preliminary dose-response experiments showing maximal superoxide stimulation without substrate inhibition. DPI concentration (10 µM) was chosen to achieve >90% inhibition of flavin-containing NADH oxidases based on published *K*_*i*_ values ^28^.

### Nitric oxide exposure and nitrogen dioxide quantification

Following treatment application, slurry headspace was flushed with synthetic air (79% N_2_, 21% O_2_; BOC) for 5 min to establish baseline conditions. Baseline headspace NO_2_ concentration was measured, then nitric oxide (45 ppb in N_2_) was introduced by replacing 50 ml of headspace with calibrated NO gas mixture via gas-tight syringe. The 45 ppb NO concentration was selected to represent the upper range of soil pore-space NO concentrations observed in agricultural soils following fertilisation^29^. It is important to note that the synthetic air carrier gas used was free of ozone, which precludes NO-to-NO_2_ conversion via NO + O_3_ reaction from impacting our results^30^. Headspace NO_2_ accumulation was monitored before NO addition, directly after, and four times over the course of 60 min using an AIRYX ICAD-HONO/NO_2_-200L analyser (Airyx GmbH, Germany). Measurement vessels were covered in aluminium foil during the experiment to exclude unintended photolytic effects. NO_2_ production rates were calculated as the slope of linear regression of headspace concentration versus time, expressed as ppb h^-1^. The instrument employs incoherent broadband cavity-enhanced absorption spectroscopy (IBBCEAS) with iterative spectral fitting, providing direct (absolute) NO_2_ measurement without chemical conversion or interference from other nitrogen oxides^31^. The optical path length was 2.4 km (effective), achieved through ~4800 reflections in a 0.5 m cavity with mirrors of 99.99% reflectivity. Spectra were acquired at 1 Hz and averaged to 10-second intervals for analysis. Baseline headspace NO concentrations were recorded prior to NO addition and were consistently below 6.5 ppb across all treatments, confirming that endogenous soil NO production did not contribute measurably to headspace NO_2_ accumulation under the sealed slurry conditions used here. Concurrent monitoring of headspace NO throughout the incubation confirmed that the NO trajectory was consistent with consumption of the introduced spike, with no evidence of sustained endogenous NO accumulation that would inflate apparent conversion rates.

### Extracellular superoxide quantification

Extracellular superoxide (O_2_⁻) was quantified by hydroethidine (HE) oxidation, with ferricytochrome c reduction run in parallel as an independent qualitative check. Because superoxide is short-lived in aqueous buffer at neutral pH, these assays do not recover a preserved standing pool; rather, they report the SOD-inhibitable superoxide-generating activity of the cell-free soil extract measured during the assay incubation. The aqueous extraction used here therefore represents a modification of DMSO-based soil superoxide protocols^32^, which target mineral-associated superoxide preserved under anhydrous conditions. Soil extracts were prepared from the end-timepoint samples by adding 10 ml ice-cold phosphate buffer (50 mM, pH 7.4, containing 100 µM diethylenetriaminepentaacetic acid [DTPA] to chelate transition metals) to 5 g soil. Suspensions were vortexed for 2 minutes, centrifuged at 5000 x g for 10 min at 4 °C, and supernatants filtered through 0.45 µm cellulose acetate filters (Whatman). Filtered extracts were held on ice and assayed without delay, and all superoxide values were derived from the SOD-inhibitable signal to control for non-superoxide reactions arising during processing and incubation. Per-well values were corrected for the extraction dilution (5 g soil into 10 ml buffer, 100 µL assayed) and normalised to the dry mass of soil to give pmol O_2_¯ g^−1^ soil. The filtrate represents the soluble, filterable extracellular superoxide-generating activity. For the hydroethidine assay, soil extract (100 µL) was combined with hydroethidine (10 µL of 10 mM stock in DMSO; final concentration 1 mM; ABCR UK LTD, ≥95% purity) in black 96-well microplates. Parallel wells received superoxide dismutase (SOD; 500 U ml^−1^; Sigma-Aldrich, ≥95% by biuret). Plates were incubated for 15 min at 25 °C in darkness, and fluorescence was measured at λex = 480 nm, λem = 580 nm using a FLUOstar Omega (BMG Labtech) microplate reader^33^. Extracts and plates were protected from light throughout, as hydroethidine is photosensitive. Because bulk hydroethidine fluorescence at these wavelengths is not fully specific to the superoxide product 2-hydroxyethidium (2-OH-E^+^), only the SOD-inhibitable fraction was taken as superoxide-derived, and values are reported and interpreted on that basis. The SOD-inhibitable signal was converted to superoxide using a calibration curve generated with the xanthine/xanthine oxidase system described below, run in the same plate format. For the cytochrome c assay, soil extract (200 µL) was added to ferricytochrome c (50 µM final; from bovine heart, Sigma-Aldrich, ≥95% by molecular weight) in clear 96-well plates, and reduction was monitored at 550 nm over 10 min; the SOD-inhibitable fraction was determined from parallel wells containing 500 U ml^-1^ SOD. Superoxide was quantified from the initial linear rate of SOD-inhibitable reduction against a standard curve prepared in the same plate format using superoxide generated by xanthine (50 µM) / xanthine oxidase (0.01 U ml^-1^), with the flux of the standard defined by the rate of cytochrome c reduction using Δε_550_ = 21.1 mM^-1^ cm^-1^. Calibrating in-plate avoids reliance on a fixed 1 cm path length in the microplate geometry. For the hydroethidine assay, superoxide was quantified from the SOD-inhibitable fluorescence at the 15-minute endpoint and is the quantity reported throughout; for the cytochrome c assay it was quantified from the initial linear rate of SOD-inhibitable reduction. Both were expressed on a common basis of pmol O_2_⁻ g^-1^ soil generated over the 15-minute assay window. The xanthine/xanthine oxidase standard was run over the same 15-minute window, so endpoint fluorescence was related to a known amount of superoxide generated over that time. Detection limits, defined as 3σ of the assay blank, were 9 pmol g^-1^ for the hydroethidine assay and 36 pmol g^-1^ for cytochrome c. Each sample was assayed in duplicate wells; the assay blank was buffer plus hydroethidine without extract, and all endpoint values fell within the linear range of the standard curve. As many of the sterile and NADH + DPI samples fell below the cytochrome c detection limit, superoxide was quantified solely by the hydroethidine assay. Cytochrome c reduction values qualitatively corroborated the treatment-level trend.

### Superoxide dismutase scavenging experiments

To directly test the requirement for superoxide in NO_2_ formation, exogenous superoxide dismutase (SOD) was added to scavenge O_2_¯ before it could react with NO. SOD from bovine erythrocytes (Sigma-Aldrich, ≥3000 U mg^−1^ protein) was dissolved in phosphate buffer (50 mM, pH 7.4) and added to slurries at a final concentration of 500 U ml^−1^. This concentration was selected to achieve complete superoxide scavenging based on the measured superoxide production rates and SOD kinetics (rate constant of k = 2 × 10^9 ^M^−1^ s^−1^). Heat-inactivated SOD controls were prepared by incubating SOD solution (1000 U ml^−1^) at 100°C for 10 minutes, which eliminates enzymatic activity. Complete inactivation was verified by superoxide methods described above. Comparison of active versus heat-inactivated SOD effects distinguished enzymatic superoxide scavenging from potential non-specific effects of protein addition (e.g., NO binding, surface interactions). SOD was added to slurries 5 min before NO introduction. NO_2_ production rates were measured as described above, and the SOD-sensitive fraction was calculated as shown in Eq. 1,

1 $${{NO}2}_{{SOD}}=\,\frac{{Rate}\,{without}\,{SOD}-{Rate}\,{with}\,{SOD}}{{Rate}\,{without}\,{SOD}}\,\times \,100$$

### Peroxynitrite synthesis and addition experiments

Peroxynitrite (ONOO¯) was synthesised by the reaction of acidified hydrogen peroxide with sodium nitrite, following established methods^34^. Briefly, ice-cold solutions of NaNO_2_ (0.6 M) and H_2_O_2_ (0.6 M in 0.7 M HCl) were mixed rapidly using a quenched-flow reactor, and the product immediately quenched with excess NaOH (1.5 M) to yield alkaline peroxynitrite stock (~180 mM). Concentration was determined spectrophotometrically at 302 nm. Stocks were stored at −80 °C in small aliquots and used within 2 weeks. Purity was verified by the absence of significant absorbance at 350 nm (indicating minimal nitrite contamination). For dose-response experiments, sterile soil (200 g) was placed in fluoropolymer-coated PVC microcosms (~0.6 L) with continuous headspace monitoring. Peroxynitrite was diluted in ice-cold NaOH (10 mM) immediately before use and applied to the soil surface at concentrations of 0 (NaOH vehicle), 0.08, 0.16, and 0.32 µmol (per gram soil). Headspace total reactive nitrogen oxides (NO_z_ = NO_y_ − NO_x_) were monitored for 60 minutes using a Teledyne T200U-NO_y_ chemiluminescence analyser. The T200U employs molybdenum catalytic conversion at 325 °C for NO_y_ measurement and direct chemiluminescence for NO. For field-fresh soil validation experiments, soil slurries (5 g soil in 15 ml deionised water) in 50 ml serum vials were amended with either 0.1 μmol g^-1^ ONOO¯ or vehicle (NaOH) and incubated at 25 °C with orbital shaking (150 rpm) for 8 h. Headspace was sampled at 8 h and analysed for NO, NO_2_, and NO_z_ by chemiluminescence.

### Temperature response experiments

To assess the temperature dependence of biogenic NO_2_ production, slurry incubations were conducted at 5°C, 15°C, and 30°C in temperature-controlled incubators (±0.5°C). Slurries were equilibrated at the target temperature for 2 hours before NO addition and measurements.

### Nitric oxide consumption and conversion efficiency

To quantify the stoichiometry of NO-to-NO_2_ conversion, headspace NO was monitored simultaneously with NO_2_. NO consumption was calculated as the decrease in headspace NO concentration over the first 60 min of the incubation, the interval over which the NO spike was actively consumed. Conversion efficiency was calculated as shown in Eq. 2,

2 $${{CE}}_{{NO}\to {NO}2}=\left(\frac{{NO}2\,{produced}}{{NO}\,{consumed}}\right)\times 100$$

The theoretical maximum for the superoxide mechanism is 100% (e.g., 1 NO + 1 O_2_⁻ → 1 ONOO⁻ → 1 NO_2_), though competing reactions reduce observed efficiency. As noted above, NO-to-NO_2_ conversion is not due to NO oxidation by ozone, since its concentration is negligible under the conditions of our experiments.

### Statistical analysis

All statistical analyses were performed in R (version 4.3.0), with data visualised using Python (version 3.12) and Adobe Illustrator (version 28.0). Data were tested for normality (Shapiro-Wilk test) and homogeneity of variance (Levene’s test) before parametric analyses. Treatment effects on NO_2_ production rates (Fig. 1A) were assessed by two-sided two-sample t-tests, where each treatment was compared with the sterile control within each soil, and NADH was compared with NADH + DPI with data pooled across both soils. The effect of SOD on NO_2_ production (Fig. 2A) was assessed by paired *t*-test, pairing each SOD-treated vessel with the untreated (Fig. 1A) vessel prepared from the same biological soil sample, within each soil and treatment. Active and heat-inactivated SOD were each compared to the no-SOD control by two-sample *t-*test (Fig. 2B). Peroxynitrite dose effects were assessed by one-way ANOVA with Tukey’s HSD post-hoc tests against the 0 µmol g^−1^ baseline (Fig. 2C), and +ONOO¯ versus −ONOO¯ headspace concentrations by two-sample *t-*test (Fig. 2D). The relationship between NO consumed and NO_2_ produced was assessed by linear regression (Fig. 1C). Correlations between superoxide concentration and NO_2_ production were assessed using Pearson’s product-moment correlation coefficient. Temperature effects were analysed using two-way ANOVA with temperature and treatment as factors. Statistical significance was set at α = 0.05. Effect sizes were calculated as Cohen’s d for pairwise comparisons. Uncertainties reported in the text are standard deviations. Error bars in all figures are S.E.M., and box plots are defined in the corresponding legends. Individual data points are overlaid on all summary statistics to display the data distribution.

## Supplementary information

Supplementary Information

Peer Review file

## Data Availability

The NO_2_, NO, superoxide and peroxynitrite source data generated in this study have been deposited in a Zenodo repository under accession code https://doi.org/10.5281/zenodo.21771492.

## Code availability

The R and Python code for statistical and data visualisation, have been deposited in a Zenodo repository under accession code https://doi.org/10.5281/zenodo.21771492.

## References

[1] Weng, H. et al. Global high-resolution emissions of soil NO_x_, sea salt aerosols, and biogenic volatile organic compounds. *Sci. Data* **7**, 148 (2020). 32433468 10.1038/s41597-020-0488-5 PMC7239948

[2] Hudman, R. C. et al. Steps towards a mechanistic model of global soil nitric oxide emissions: implementation and space based-constraints. *Atmos. Chem. Phys.* **12**, 7779–7795 (2012).

[3] Lee, B. H. et al. Sensitive response of atmospheric oxidative capacity to the uncertainty in the emissions of nitric oxide (NO) from soils in Amazonia. *Geophys. Res. Lett.* **51**, e2023GL107214 (2024).

[4] Opacka, B. et al. Natural emissions of VOC and NOx over Africa constrained by TROPOMI HCHO and NO_2_ data using the MAGRITTEv1.1 model. *Atmos. Chem. Phys.* **25**, 2863–2894 (2025).

[5] Diaz, J. M. et al. Widespread production of extracellular superoxide by heterotrophic bacteria. *Science* **340**, 1223–1226 (2013). 23641059 10.1126/science.1237331

[6] Pryor, W. A. & Squadrito, G. L. The chemistry of peroxynitrite: a product from the reaction of nitric oxide with superoxide. *Am. J. Physiol.-Lung Cell. Mol. Physiol.* **268**, L699–L722 (1995). 10.1152/ajplung.1995.268.5.L699 7762673

[7] Merényi, G., Lind, J., Goldstein, S. & Czapski, G. Mechanism and thermochemistry of peroxynitrite decomposition in water. *J. Phys. Chem. A*. **103**, 5685–5691 (1999).

[8] Huie, R. E. & Padmaja, S. The reaction of no with superoxide. *Free Radic. Res. Commun.* **18**, 195–199 (1993). 8396550 10.3109/10715769309145868

[9] Bielski, B. H. J., Cabelli, D. E., Arudi, R. L. & Ross, A. B. Reactivity of HO_2_/O^−^_2_ radicals in aqueous solution. *J. Phys. Chem. Ref. Data* **14**, 1041–1100 (1985).

[10] Goldstein, S. & Czapski, G. Formation of peroxynitrate from the reaction of peroxynitrite with CO_2_: evidence for carbonate radical production. *J. Am. Chem. Soc.* **120**, 3458–3463 (1998).

[11] Augusto, O. et al. Carbon dioxide-catalyzed peroxynitrite reactivity – The resilience of the radical mechanism after two decades of research. *Free Radic. Biol. Med.* **135**, 210–215 (2019). 30818056 10.1016/j.freeradbiomed.2019.02.026

[12] Jensen, M. P. & Riley, D. P. Peroxynitrite decomposition activity of iron porphyrin complexes. *Inorg. Chem.* **41**, 4788–4797 (2002). 12206706 10.1021/ic011089s

[13] Szabó, C., Ischiropoulos, H. & Radi, R. Peroxynitrite: biochemistry, pathophysiology and development of therapeutics. *Nat. Rev. Drug Discov.* **6**, 662–680 (2007). 17667957 10.1038/nrd2222

[14] Kebede, M. A., Bish, D. L., Losovyj, Y., Engelhard, M. H. & Raff, J. D. The role of iron-bearing minerals in NO_2_ to HONO conversion on soil surfaces. *Environ. Sci. Technol.* **50**, 8649–8660 (2016). 27409359 10.1021/acs.est.6b01915

[15] Scharko, N. K., Martin, E. T., Losovyj, Y., Peters, D. G. & Raff, J. D. Evidence for quinone redox chemistry mediating daytime and nighttime NO_2_-to-HONO conversion on soil surfaces. *Environ. Sci. Technol.* **51**, 9633–9643 (2017). 28742971 10.1021/acs.est.7b01363

[16] Qiu, Y. et al. Intermediate soil acidification induces highest nitrous oxide emissions. *Nat. Commun.* **15**, 2695 (2024). 38538640 10.1038/s41467-024-46931-3 PMC10973416

[17] Frostegård, Å, Vick, S. H. W., Lim, N. Y. N., Bakken, L. R. & Shapleigh, J. P. Linking meta-omics to the kinetics of denitrification intermediates reveals pH-dependent causes of N_2_O emissions and nitrite accumulation in soil. *ISME J.* **16**, 26–37 (2022). 34211102 10.1038/s41396-021-01045-2 PMC8692524

[18] Leitner, S. et al. Linking NO and N_2_O emission pulses with the mobilization of mineral and organic N upon rewetting dry soils. *Soil Biol. Biochem.* **115**, 461–466 (2017).

[19] Mushinski, R. M. et al. Microbial mechanisms and ecosystem flux estimation for aerobic NOy emissions from deciduous forest soils. *Proc. Natl. Acad. Sci.* 201814632 10.1073/pnas.1814632116 (2019). PMC6369794 30659144

[20] Williams, E. J., Parrish, D. D. & Fehsenfeld, F. C. Determination of nitrogen oxide emissions from soils: Results from a grassland site in Colorado, United States. *J. Geophys. Res. Atmos.* **92**, 2173–2179 (1987).

[21] Shah, M. et al. Modification and validation of a commercial dynamic chamber for reactive nitrogen and greenhouse gas flux measurements. *Atmos. Meas. Tech.* **19**, 2379–2405 (2026).

[22] Boys, B. L., Martin, R. V. & VandenBoer, T. C. Evaluation of and updates to the oxidized reactive nitrogen gaseous dry-deposition parameterization from the GEOS-Chem model, including a pathway for ground surface NO2 hydrolysis. *Atmos. Chem. Phys.* **25**, 17553–17580 (2025).

[23] McDonald, M. D. et al. Nitrogen fertilizer driven nitrous and nitric oxide production is decoupled from microbial genetic potential in low carbon, semi-arid soil. *Front. Soil Sci.* **2**, 1050779 (2023).

[24] Ludwig, J., Meixner, F. X., Vogel, B. & Förstner, J. Soil-air exchange of nitric oxide: an overview of processes, environmental factors, and modeling studies. *Biogeochemistry* **52**, 225–257 (2001).

[25] Parton, W. J. et al. Generalized model for NO_x_ and N_2_O emissions from soils. *J. Geophys. Res. Atmos.* **106**, 17403–17419 (2001).

[26] Val Martin, M. et al. Improving nitrogen cycling in a land surface model (CLM5) to quantify soil N_2_O, NO, and NH_3_ emissions from enhanced rock weathering with croplands. *Geosci. Model Dev.* **16**, 5783–5801 (2023).

[27] Wolf, D. C., Dao, T. H., Scott, H. D. & Lavy, T. L. Influence of sterilization methods on selected soil microbiological, physical, and chemical properties. *J. Environ. Qual.* **18**, 39–44 (1989).

[28] O’Donnell, V. B., Tew, D. G., Jones, O. T. G. & England, P. J. Studies on the inhibitory mechanism of iodonium compounds with special reference to neutrophil NADPH oxidase. *Biochem. J.* **290**, 41–49 (1993). 8439298 10.1042/bj2900041 PMC1132380

[29] Venterea, R. T. & Rolston, D. E. Nitric and nitrous oxide emissions following fertilizer application to agricultural soil: Biotic and abiotic mechanisms and kinetics. *J. Geophys. Res. Atmos.* **105**, 15117–15129 (2000).

[30] Williams, E. J., Hutchinson, G. L. & Fehsenfeld, F. C. NO_x_ and N_2_O emissions from soil. *Glob. Biogeochem. Cycles* **6**, 351–388 (1992).

[31] Langridge, J. M., Ball, S. M. & Jones, R. L. A compact broadband cavity enhanced absorption spectrometer for detection of atmospheric NO_2_ using light emitting diodes. *Analyst* **131**, 916–922 (2006). 17028725 10.1039/b605636a

[32] Georgiou, C. D., Papapostolou, I. & Grintzalis, K. Superoxide radical detection in cells, tissues, organisms (animals, plants, insects, microorganisms) and soils. *Nat. Protoc.* **3**, 1679–1692 (2008). 18846095 10.1038/nprot.2008.155

[33] Zielonka, J., Vasquez-Vivar, J. & Kalyanaraman, B. Detection of 2-hydroxyethidium in cellular systems: a unique marker product of superoxide and hydroethidine. *Nat. Protoc.* **3**, 8–21 (2008). 18193017 10.1038/nprot.2007.473

[34] Robinson, K. M. & Beckman, J. S. in *Methods in Enzymology* Vol. 396 207-214 (Academic Press, 2005). 10.1016/S0076-6879(05)96019-9 16291234

