# Supplementary material for: Microbial superoxide production drives biogenic nitrogen dioxide formation in soils
Source: Nat Commun. 2026 Aug 17;17:9845. doi: 10.1038/s41467-026-76881-x (PMC13578383; doi:10.1038/s41467-026-76881-x)
Supplement: Supplementary file 1 [file 41467_2026_76881_MOESM1_ESM.pdf]

Supporting Information for
Microbial superoxide production drives biogenic nitrogen dioxide formation in soils

Megan L. Purchase, Jonathan D. Raff, Deying Wang, and Ryan M. Mushinski*
*Corresponding Author's Email: Ryan.Mushinski@warwick.ac.uk

Table S1. Physicochemical properties of experimental soils.

Property	Agricultural	Woodland
Texture class	Sandy loam	Clay loam
Sand (%)	60 ± 3	32 ± 4
Silt (%)	25 ± 2	35 ± 3
Clay (%)	15 ± 2	33 ± 4
pH (H_2O)	6.8 ± 0.3	5.9 ± 0.2
Organic matter (%)	3.2 ± 0.4	8.1 ± 0.6
Total C (g kg^{-1})	18.6 ± 2.1	47.2 ± 3.8
Total N (g kg^{-1})	1.9 ± 0.2	4.1 ± 0.4
C:N ratio	9.8 ± 0.5	11.5 ± 0.8
WHC (g g^{-1})	0.42 ± 0.03	0.68 ± 0.05
Available NH_4^+ (µg N g^{-1} dw)	0.59 ± 0.25	1.57 ± 0.76
Available NO_3^- (µg N g^{-1} dw)	1.87 ± 1.21	0.99 ± 0.08

Values are mean ± SD. n = 5. WHC, water holding capacity; dw, dry weight.
