# Supplementary material for: Microbial superoxide production drives biogenic nitrogen dioxide formation in soils
Source: Nat Commun. 2026 Aug 17;17:9845. doi: 10.1038/s41467-026-76881-x (PMC13578383; doi:10.1038/s41467-026-76881-x)
Supplement: Supplementary file 2 [file 41467_2026_76881_MOESM2_ESM.pdf]

nature portfolio

Peer Review File

Microbial superoxide production drives biogenic nitrogen dioxide formation in soils

Corresponding Author: Dr Ryan Mushinski

This file contains all reviewer reports in order by version, followed by all author rebuttals in order by version.

Version 0:

Reviewer comments:

Reviewer #1

(Remarks to the Author)
Line 15, Using manipulative slurry experiments. In this phrase, manipulative word looks odd, alternatives can be used for the clarity and scientific soundness. Same in line 47.
Line 39-43, it is also matter of concern that what is the link of microbial ROS in NO2 production and use of oxygen in oxygen limited conditions (anaerobic)?
Figure 1 C, y-axis title need to check the unit, ppb h-1, right? Please confirm.
Results are well explained and discussed with relevant literature. In addition, this work provides mechanistic explanation of the gap how and from where NO2 production comes from in field chambers experiment which has been often ignored in N modeling. This new pathway of NO2 production via biogenic microbial ROS provides practical implications as well for managing land, fertilizers use and climate change mitigation.
Results are well aligned with literature and support the conclusion and main findings.
Methodology is enough for the reproducibility.

Reviewer #2

(Remarks to the Author)
The authors present a clear mechanistic link between soil NO_2 emissions and microbial superoxide production. They further demonstrate that peroxynitrite acts as an intermediate in microbial superoxide formation. This novel finding highlights the complexity of the nitrogen cycle and gaseous nitrogen emissions and is highly relevant for improving our understanding of the soil NO_2 budget. The manuscript is concise and successfully supports the authors' hypothesis. Therefore, I support its publication. However, before acceptance, the following concerns should be addressed:
1. Choice of experimental setup:
Why were soil slurries used instead of homogenized soils? Please clarify the rationale and discuss how this choice may influence microbial processes and gas fluxes.
2. Baseline NO emissions:
NO was added to the headspace to ensure sufficient substrate for NO_2 production. However, soils are known to emit NO naturally. What were the baseline NO emissions from this soil, and how might they have affected the observed NO_2 production rates? Were background NO levels accounted for in the calculations?
3. Mineral nitrogen availability:
Mineral N concentrations of the soil are not reported. Were nitrate and ammonium measured? If so, please report these values. Additionally, discuss whether mineral N availability could influence the observed processes, particularly given the likely role of heterotrophic bacteria.
4. Broader relevance and implications:
Two important aspects are currently missing:
o The relevance of these findings in the context of soil physicochemical properties in general should be discussed in more detail.
o The types of ecosystems or land-use scenarios in which these processes are likely to be most important should be addressed. While this may be beyond the immediate scope of the study, it would provide valuable direction for future research.
5. NO_2 flux calculations:

The methodology for calculating NO_2 flux rates is unclear. If not already included, please provide detailed information on how these fluxes were calculated.

Reviewer #3

(Remarks to the Author)
The paper reports the results of some experiments showing that when soils are exposed to high levels of gas phase nitric oxide (NO), the presence of naturally occurring (and artificially induced) superoxide (O2-) can lead to the conversion of NO into nitrogen dioxide (NO2). This chemistry, which occurs via the formation of peroxynitrite, has been well-known in physiological systems for decades (e.g. Pryor and Squadrito, 1995), as acknowledged by the authors. The authors posit that this chemistry can also occur in soils, driven by co-existing communities of microorganisms. I found the experiments with native and sterilized soils to be convincing in terms of the explanation for the drivers of the process.

However, the paper is framed to suggest that this pathway may be important in the understanding of soil-atmosphere fluxes of nitrogen oxides, which are typically understood and represented as emissions of NO and deposition of NO2. The main interest around soil-atmosphere nitrogen oxide (NO + NO2) fluxes is motivated by their role in sustaining atmospheric radical cycling and oxidation capacity. Because NO and NO2 are rapidly interconverted by oxidants and sunlight in the atmosphere, detailed knowledge of the exact form of the emission is viewed as less important. The authors do not sufficiently connect the dots as the whether the process they observed in the lab (caused by the addition of large amounts of exogenous NO) would actually manifest in a meaningful way in the environment itself. In fact, on lines 110 – 116, the authors identify only two references where authors reported detecting evidence of direct NO2 emissions from soils in the environment. To my mind, this suggests that the mechanism may not manifest itself under natural conditions very often and undermines its proposed importance.

It is true that current biogeochemical models do not include the process of NO to NO2 conversion in soil (as stated on line 109), but most estimates of NOx emissions are driven by empirical paramterizations like the The Berkeley Dalhousie (Iowa) Soil NO Parameterization (BD-SNP) scheme (e.g. Rasool et al., 2016; Sha et al., 2021), rather than process-level biogeochemical models. It is far from obvious whether the mechanism identified by the authors would be any impact on the emissions predicted by these models.

I was also missing any reference to the pKa of peroxynitrous acid and the extent to which its formation, and potential conversion to nitrate may short circuit the production and release of NO2.

Lines 32-34 This statement glosses over dozens of references over 15 years that have described the improvements to parameterization schemes representing soil NOx emissions. Large uncertainties in the magnitudes and drivers of soil NOx emissions certainly exist, and the extent to which canopy reduction factors should be implemented, but there is little evidence that mismatches between model and observation can be explained by the mechanism invoked by the authors.

Figure 2E – this representation seems to imply that NO is not directly emitted by the soil (at all), which is certainly counter to many observations. Would be good to add a dotted green arrow.

References
Pryor WA, Squadrito GL. The chemistry of peroxynitrite: a product from the reaction of nitric oxide with superoxide. Am J Physiol. (1995) 268(5 Pt 1):L699-722. doi: 10.1152/ajplung.1995.268.5.L699.
Rasool, Q. Z. , Zhang, R. , Lash, B. , Cohan, D. S. , Cooter, E. J. , Bash, J. O. , & Lamsal, L. N. (2016). Enhanced representation of soil NO emissions in the community multiscale air quality (CMAQ) model version 5.0.2. Geoscientific Model Development, 9(9), 3177–3197. 10.5194/gmd-9-3177-2016
Sha, T., Ma, X., Zhang, H., Janechek, N., Wang, Y., Wang, Y., Castro Garci´a, L., Jenerette, G. D., and Wang, J.: Impacts of Soil NOx Emission on O3 Air Quality in Rural California, Environ. Sci. Technol., 55, 7113–7122, 2021.

Open Access This Peer Review File is licensed under a Creative Commons Attribution 4.0 International License, which permits use, sharing, adaptation, distribution and reproduction in any medium or format, as long as you give appropriate credit to the original author(s) and the source, provide a link to the Creative Commons license, and indicate if changes were made.
In cases where reviewers are anonymous, credit should be given to 'Anonymous Referee' and the source.
The images or other third party material in this Peer Review File are included in the article's Creative Commons license, unless indicated otherwise in a credit line to the material. If material is not included in the article's Creative Commons license and your intended use is not permitted by statutory regulation or exceeds the permitted use, you will need to obtain permission directly from the copyright holder.
To view a copy of this license, visit https://creativecommons.org/licenses/by/4.0/

Reviewer 1

Reviewer 1, Comment 1: Line 15, Using manipulative slurry experiments. In this phrase, manipulative word looks odd, alternatives can be used for the clarity and scientific soundness. Same in line 47.

> **Response**: We agree. “Manipulative” has been replaced with “controlled factorial” throughout the abstract and Methods section. The abstract now reads, “Using controlled factorial slurry experiments, native microbial communities produced 6-10 times more NO_2 than sterile controls...”

Reviewer 1, Comment 2: Line 39-43, it is also matter of concern that what is the link of microbial ROS in NO2 production and use of oxygen in oxygen limited conditions (anaerobic)?

> **Response:** This is an important clarification. Extracellular superoxide production by heterotrophic NADH oxidases is an inherently aerobic process, as molecular oxygen serves as the terminal electron acceptor for the oxidoreductase enzymes responsible for O_2^- generation. The proposed mechanism therefore requires aerobic conditions and would not operate under anaerobic or strongly oxygen-limited conditions. We have added the following sentence to the Results section to make this explicit, “This superoxide-driven pathway is inherently aerobic, as extracellular O_2^- production by heterotrophic NADH oxidases requires molecular oxygen as the terminal electron acceptor. The mechanism is therefore expected to be most significant in surface and near-surface soil horizons where aerobic conditions prevail, and biogenic NOx emissions occur.” We note that this is consistent with the broader soil NOx literature, which treats biogenic NO emissions as predominantly aerobic surface processes. The mechanism described here occupies the same environmental niche.

Reviewer 1, Comment 3: Figure 1C, y-axis title need to check the unit, ppb h-1, right? Please confirm.

> **Response:** We thank the reviewer for flagging this. To clarify, the y-axis of Figure 1C shows cumulative NO_2 produced (ppb), not a rate (ppb h^{-1}). This panel is a stoichiometric conversion efficiency plot, showing the relationship between total NO consumed and total NO2 produced over the first 60 minutes of the incubation. The unit of ppb (cumulative concentration) is therefore correct for this panel. The axis label in the figure reads “NO_2 produced (ppb)” and is accurate as shown.

Reviewer 2

Reviewer 2, Comment 1: The authors present a clear mechanistic link between soil NO2 emissions and microbial superoxide production. They further demonstrate that peroxynitrite acts as an intermediate in microbial superoxide formation. This novel finding highlights the complexity of the nitrogen cycle and gaseous nitrogen emissions and is highly relevant for improving our understanding of the soil NO2 budget. The manuscript is concise and successfully supports the authors' hypothesis. Therefore, I support its publication. However, before acceptance, the following concerns should be addressed.

Response: We thank Reviewer 2 for their supportive assessment and constructive comments. We address each point below.

Reviewer 2, Comment 2: Why were soil slurries used instead of homogenized soils? Please clarify the rationale and discuss how this choice may influence microbial processes and gas fluxes.

Response: We have added a dedicated paragraph to the Slurry Incubation Experiments section of the Methods explaining the rationale and acknowledging the limitation. The following text was added, "Aerobic slurries were selected over homogenised dry soils to ensure homogeneous distribution of chemical amendments throughout the soil matrix, which we found essential for the mechanistic inhibition and stimulation experiments central to this study, and to provide a sufficient aqueous phase for superoxide quantification via the hydroethidine and cytochrome c reduction assays. We acknowledge that slurry conditions may enhance diffusion of reactive species relative to intact soil, potentially increasing measured NO_2 production rates relative to field conditions; however, the primary purpose of these experiments was mechanistic demonstration rather than quantitative flux estimation, and the relative differences between treatments are robust to this consideration." The intent is to convey that the experimental design was optimised for mechanistic resolution rather than flux quantification, since the selective enzyme inhibitors (DPI) and scavengers (SOD) required homogeneous reagent distribution that is not achievable in intact soil cores.

Reviewer 2, Comment 3: NO was added to the headspace to ensure sufficient substrate for NO2 production. However, soils are known to emit NO naturally. What were the baseline NO emissions from this soil, and how might they have affected the observed NO2 production rates? Were background NO levels accounted for in the calculations?

Response: This is an important methodological point. We have added the following text to the end of the Nitric Oxide Exposure and Nitrogen Dioxide Quantification section. "Baseline headspace NO concentrations were recorded prior to NO addition and were consistently below 6.5 ppb across all treatments, confirming that endogenous soil NO production did not contribute measurably to headspace NO_2 accumulation under the sealed slurry conditions used here. Concurrent monitoring of headspace NO throughout the incubation confirmed that the NO trajectory was consistent with consumption of the introduced spike, with no evidence of sustained endogenous NO accumulation that would inflate apparent conversion rates." The low baseline NO values reflect the fact that the slurry headspace was flushed with synthetic air for 5 minutes prior to the baseline measurement, purging any accumulated soil-produced NO. The 45 ppb NO spike introduced thereafter represented the primary NO substrate for the conversion experiments. The concurrent NO monitoring throughout the incubation (captured in the Fig. 1C stoichiometry data) confirms that the NO dynamics are fully consistent with substrate consumption rather than ongoing endogenous production.

Reviewer 2, Comment 4: Mineral N concentrations of the soil are not reported. Were nitrate and ammonium measured? If so, please report these values. Additionally, discuss whether mineral N availability could influence the observed processes, particularly given the likely role of heterotrophic bacteria.

Response: Available NH_4^+ and NO_3^- concentrations have been measured and are now reported in Table S1 of the Supporting Information. Values are, agricultural soil, NH_4^+ = 0.59 ± 0.25 μg N g^{-1} dw, NO_3^- = 1.87 ± 1.21 μg N g^{-1} dw; woodland soil, NH_4^+ = 1.57 ± 0.76 μg N g^{-1} dw, NO_3^- = 0.99 ± 0.08 μg N g^{-1} dw. We have also added interpretation of these values to the Discussion. "The higher NO_3^- availability in the agricultural soil (Table S1) is consistent with greater nitrification activity and NO substrate supply, while the higher NH_4^+ and organic matter content of the woodland soil supports greater heterotrophic superoxide production capacity. That both soils converge on similar NO_2 production rates despite these contrasting properties suggests the mechanism is robust across a range of soil biogeochemical conditions where the two requirements are met through different combinations of soil properties." These data support the co-dependence of the mechanism on NO substrate availability (favoured by nitrification in the higher-NO_3^- agricultural soil) and heterotrophic superoxide production capacity (favoured by the higher organic matter and NH_4^+ in the woodland soil).

Reviewer 2, Comment 5: Two important aspects are currently missing; (1) The relevance of these findings in the context of soil physicochemical properties in general should be discussed in more detail; (2) The types of ecosystems or land-use scenarios in which these processes are likely to be most important should be addressed. While this may be beyond the immediate scope of the study, it would provide valuable direction for future research.

Response: We have substantially expanded the Discussion to address both points. A new paragraph now identifies the soil physicochemical conditions favouring the mechanism from first principles and specifies the three land-use contexts where it is expected to be most significant. The following text was added, "Circumneutral to slightly acidic pH (approximately 5.5-7.5) favours both nitrification and denitrification, ensuring the NO substrate supply necessary for the mechanism, while also maintaining the ONOOH:ONOO⁻ speciation that enables parallel decomposition pathways. Adequate aeration is a prerequisite given the aerobic nature of the superoxide-producing enzymes involved. In terms of land-use scenarios, the mechanism is therefore likely to be most significant in three contexts: fertilised agricultural soils following nitrogen application, where simultaneous stimulation of nitrifier activity and heterotrophic decomposition of organic amendments creates the necessary co-occurrence of NO and superoxide production; organic-rich forest soils where high heterotrophic activity is sustained year-round and nitrogen cycling supports background NO production; and soils experiencing wet-dry transition events, where rewetting pulses transiently stimulate both microbial communities simultaneously. Conversely, highly acidic soils (pH < 5), waterlogged anaerobic conditions, or soils with very low organic matter content would be expected to suppress the mechanism."

Reviewer 2, Comment 6: The methodology for calculating NO_2 flux rates is unclear. If not already included, please provide detailed information on how these fluxes were calculated.

Response: We have clarified this in the Methods. The Nitric Oxide Exposure and Nitrogen Dioxide Quantification section now explicitly states, "NO_2 production rates were calculated as the slope of linear regression of headspace concentration versus time, expressed as ppb h^{-1}." The instrument used

(AIRYX ICAD-HONO/NO2-200L) employs incoherent broadband cavity-enhanced absorption spectroscopy (IBBCEAS) providing direct absolute NO_2 measurement; full instrument specifications are provided in the Methods section.

Reviewer 3

Reviewer 3, Comment 1: The paper reports the results of some experiments showing that when soils are exposed to high levels of gas phase nitric oxide (NO), the presence of naturally occurring (and artificially induced) superoxide (O_2^-) can lead to the conversion of NO into nitrogen dioxide (NO_2). This chemistry, which occurs via the formation of peroxynitrite, has been well-known in physiological systems for decades (e.g. Pryor and Squadrito, 1995), as acknowledged by the authors. The authors posit that this chemistry can also occur in soils, driven by co-existing communities of microorganisms. I found the experiments with native and sterilized soils to be convincing in terms of the explanation for the drivers of the process.

Response: We thank Reviewer 3 for acknowledging the strength of the mechanistic experiments and for the detailed and substantive comments that follow. We address each point in turn.

Reviewer 3, Comment 2: However, the paper is framed to suggest that this pathway may be important in the understanding of soil-atmosphere fluxes of nitrogen oxides, which are typically understood and represented as emissions of NO and deposition of NO_2. The main interest around soil-atmosphere nitrogen oxide (NO + NO_2) fluxes is motivated by their role in sustaining atmospheric radical cycling and oxidation capacity. Because NO and NO_2 are rapidly interconverted by oxidants and sunlight in the atmosphere, detailed knowledge of the exact form of the emission is viewed as less important. The authors do not sufficiently connect the dots as to whether the process they observed in the lab (caused by the addition of large amounts of exogenous NO) would actually manifest in a meaningful way in the environment itself. In fact, on lines 110-116, the authors identify only two references where authors reported detecting evidence of direct NO_2 emissions from soils in the environment. To my mind, this suggests that the mechanism may not manifest itself under natural conditions very often and undermines its proposed importance.

Response: We thank the reviewer for this central critique and agree that the environmental relevance argument required strengthening. We have made several substantive additions to address this concern.

First, on the scarcity of field observations of direct soil NO_2 emission, the reviewer correctly notes that we cited only two such references. We have now added a third, Shah et al. (2026, Atmos. Meas. Tech.) who confirmed positive NO_2 fluxes from agricultural soils under realistic moisture conditions using a validated dynamic chamber system, explicitly noting that the scarcity of such measurements in the literature reflects methodological limitations rather than the absence of the process. Furthermore, we note that the scarcity of above-canopy observations of soil NO_2 emission is itself mechanistically explicable. Boys et al. (2025, Atmos. Chem. Phys.) recently demonstrated that canopy reduction factors of 47-59% apply to soil-emitted NO at forest sites, with this attenuation driven by NO_2 heterogeneous hydrolysis on canopy and ground surfaces. Since NO_2 is substantially more reactive

than NO toward surface chemistry, biogenic NO_2 produced in soil would be intercepted even more efficiently by the canopy and soil surfaces before reaching above-canopy measurement heights, converting to HONO via the well-characterised iron mineral and phenol pathways. The scarcity of above-canopy NO_2 flux observations is therefore a predictable consequence of rapid near-surface interception rather than evidence that the mechanism rarely operates. This argument is now developed explicitly in the Discussion.

Second, on the environmental realism of 45 ppb NO, the reviewer implies this concentration is unrealistically high. We have addressed this directly in the Discussion, citing McDonald et al. (2023, Frontiers in Soil Science), who reported average growing-season soil pore-space NO concentrations of up to 3,300 ppb in fertilised agricultural soils, and Ludwig et al. (2001, Biogeochemistry), who noted that surface ambient NO concentrations of 170 ppb recorded over fertilised soils substantially underestimate subsurface pore-space concentrations due to diffusion and dilution. The 45 ppb NO used in our experiments therefore represents a conservative, lower-bound estimate of the substrate concentrations that NO-producing soil microorganisms experience. We also note that unpublished field chamber measurements from a fertilised agricultural soil (VandenBoer, T.C., personal communication, **Response Figure 1**) recorded NO concentrations of 100-200 ppb within closed dynamic chambers following urea application at ~30 kg N ha^{-1}, substantially exceeding the 45 ppb used in our experiments. While these data are not yet peer-reviewed and are therefore not included in the manuscript itself, they provide independent confirmation that the substrate concentrations used in our laboratory experiments are environmentally conservative rather than unrealistically high.

Third, regarding the rapid interconversion of NO and NO_2 in the atmosphere and whether NOx speciation matters, the reviewer is correct that above-canopy NO and NO_2 interconvert rapidly. However, the significance of biogenic NO_2 production operates at two scales that are decoupled from above-canopy chemistry. At the soil surface, NO_2 is subject to heterogeneous hydrolysis to HONO on soil mineral and organic surfaces, a conversion that does not occur for NO and that represents a distinct pathway to atmospheric HONO with implications for daytime OH radical production. At the ecosystem scale, NO_2 produced in soil and converted to HONO below the canopy represents a source term that is entirely absent from current model parameterisations, which treat soil-atmosphere HONO exchange as exclusively abiotic. These points are now developed in the Discussion.

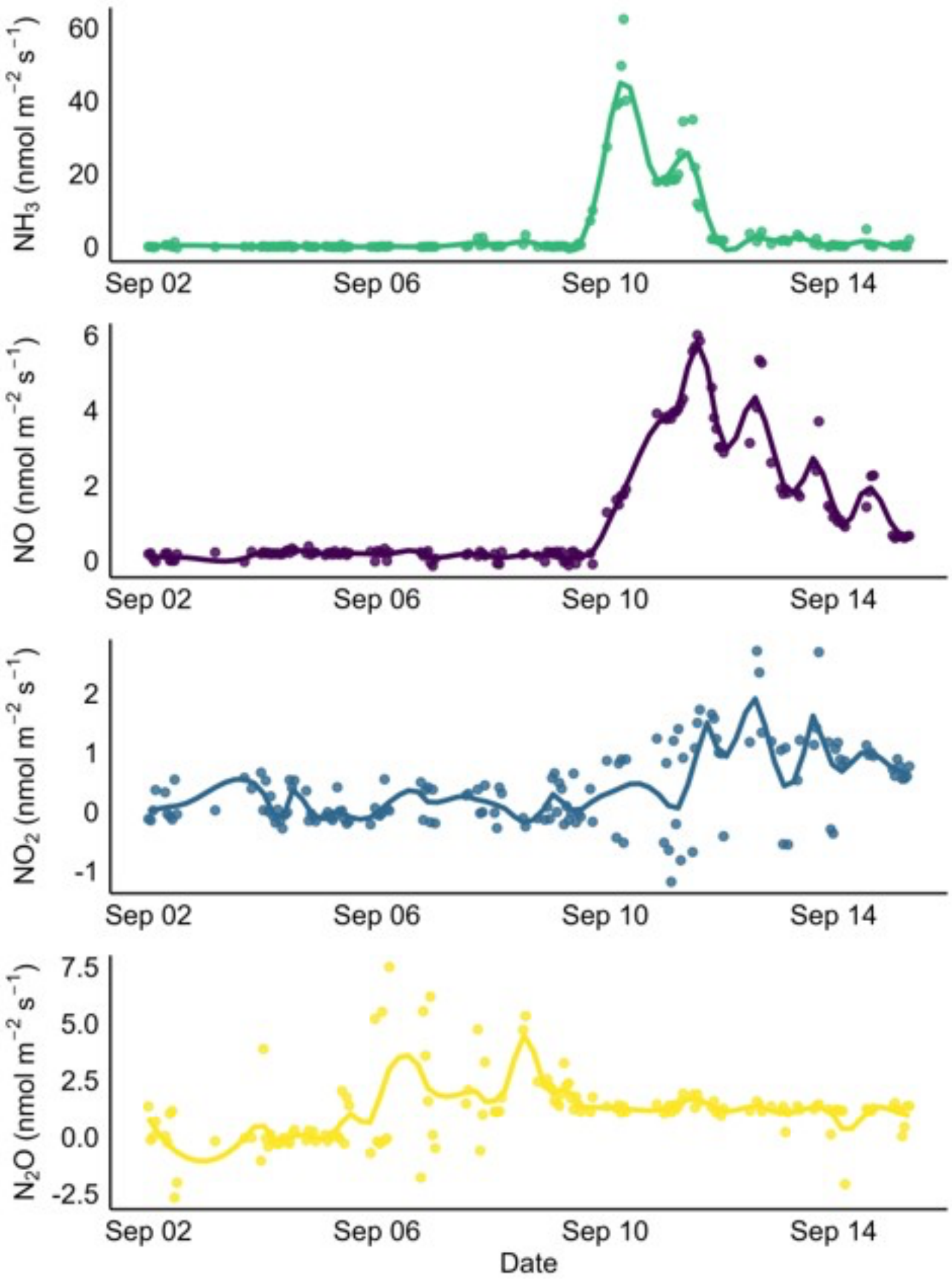

Response Figure 1. Time series of reactive nitrogen and greenhouse gas fluxes measured above a fertilised agricultural soil using a closed dynamic chamber system following urea application (~30 kg N ha^{-1}). Panels show fluxes of NH_3 (green, top), NO (purple), NO_2 (blue), and N_2O (yellow, bottom), expressed in nmol m^{-2} s^{-1}. Individual chamber closure measurements are shown as points; the solid line represents a smoothed trend. NO concentrations within the chamber headspace reached 100-200 ppb during peak flux periods (Sept. 11), substantially exceeding the 45 ppb NO used in our laboratory slurry experiments and demonstrating that soil pore-space NO concentrations under active microbial conditions are environmentally conservative rather than unrealistically high.

Reviewer 3, Comment 3: It is true that current biogeochemical models do not include the process of NO to NO_2 conversion in soil (as stated on line 109), but most estimates of NOx emissions are driven by empirical parameterizations like the Berkeley Dalhousie (Iowa) Soil NO Parameterization (BD-SNP) scheme (e.g. Rasool et al., 2016; Sha et al., 2021), rather than process-level biogeochemical models. It is far from obvious whether the mechanism identified by the authors would be any impact on the emissions predicted by these models.

Response: The reviewer raises a fair and important point. We fully agree that empirically parameterised schemes such as BD-SNP dominate operational soil NOx emission inventories, and that the mechanism described here does not translate directly into a modification of those frameworks. BD-SNP and related schemes predict total NOx emission as a function of temperature, soil moisture,

and nitrogen availability using empirically derived relationships, without representing the underlying microbial mechanisms that govern NOx speciation.

We have revised the Discussion to make this distinction explicit. The following text was added, "While direct incorporation into empirically parameterised NOx inventories will require further field-scale quantification of the NO_2:NO ratio under varying soil biogeochemical conditions, this mechanism warrants representation in process-based Earth system models and may account for a component of the HONO emissions increasingly reported from soil surfaces globally."

The more tractable near-term implementation target is the process-based modelling frameworks (CLM5, ELM, the Parton-family models) that already track the state variables (heterotrophic respiration rate, organic matter content, soil moisture, and inorganic nitrogen) needed to represent extracellular superoxide production. These models currently treat NO_2:NO ratios at the soil-atmosphere interface as fixed by assumed atmospheric chemistry rather than soil biological activity, and it is this representation that the mechanism described here most directly informs. We have clarified this framing in the revised Discussion and acknowledge that the pathway to incorporation into BD-SNP-style empirical schemes requires field-scale flux partitioning data that are not yet available.

Reviewer 3, Comment 4: I was also missing any reference to the pKa of peroxynitrous acid and the extent to which its formation, and potential conversion to nitrate may short circuit the production and release of NO2.

Response: This is an important mechanistic point that we had not addressed adequately in the original submission. We have now added a substantive treatment of peroxynitrite chemistry in soil to the Discussion.

"At our bulk soil pH values of 5.9 and 6.8, peroxynitrous acid (ONOOH; pKa 6.8) predominates or co-exists with the anion in approximately equal proportions (88:12 and 50:50 ONOOH:$ONOO^-$, respectively). Although the major terminal product of ONOOH decomposition is nitrate, NO_2 and hydroxyl radical (•OH) are obligate intermediates, with nitrate formed only when NO_2 and •OH recombine within the solvent cage. In soil, abundant organic matter and bicarbonate function as competitive •OH scavengers, kinetically hindering cage recombination and increasing the probability that NO_2 escapes into the gas phase before reacting with •OH. Additionally, $ONOO^-$ reacts with dissolved CO_2 to form the adduct $ONOOCO_2^-$, which decomposes to yield NO_2 and carbonate radical anion ($CO_3^{\bullet -}$) in approximately 30-40% yield. Given that soil CO_2 concentrations substantially exceed those of dilute aqueous systems due to active respiration, this pathway likely operates toward the higher end of this yield range in soil, representing an additional source of NO_2 that operates independently of bulk pH speciation. Fe and Mn redox-active minerals abundant in soil have also been shown to decompose peroxynitrite via one-electron oxidation pathways that generate NO_2, consistent with the established role of iron minerals in soil reactive nitrogen chemistry. Together, these pathways explain why NO_2 production is inhibited but not abolished by SOD addition, and why the mechanism is expected to remain operative across the range of pH conditions encountered in natural soils."

We have also added Pryor and Squadrito (1995), the reference specifically identified by the reviewer, to the introductory paragraph describing the physiological peroxynitrite chemistry (now ref 6 in the revised manuscript), alongside Merényi et al. (1999) and Huie and Padmaja (1993).

Reviewer 3, Comment 5: Lines 32-34 This statement glosses over dozens of references over 15 years that have described the improvements to parameterization schemes representing soil NOx emissions. Large uncertainties in the magnitudes and drivers of soil NOx emissions certainly exist, and the extent to which canopy reduction factors should be implemented, but there is little evidence that mismatches between model and observation can be explained by the mechanism invoked by the authors.

Response: The reviewer is correct that the original framing was too strong and did not adequately acknowledge the substantial body of work improving empirical soil NOx parameterisations. We have revised the introduction accordingly. The following text was added, "While empirical parameterisation schemes have improved considerably over recent decades, process-level understanding of soil NOx speciation, particularly the conditions governing NO_2 versus NO emission, remains limited."

We do not claim that the superoxide-driven pathway explains the full magnitude of satellite-model discrepancies, nor that it invalidates the progress made in empirical parameterisation. Rather, we identify it as a previously unrecognised process-level pathway that is absent from current mechanistic representations and that, if operating at field scale, would contribute a biogenic NO_2 source term not captured by any current approach. We have been careful in the revised Discussion to frame the implications in terms of process-based models rather than empirical inventories, and to acknowledge that field-scale quantification is a necessary next step.

Reviewer 3, Comment 6: Figure 2E; this representation seems to imply that NO is not directly emitted by the soil (at all), which is certainly counter to many observations. Would be good to add a dotted green arrow.

Response: We agree entirely. A dashed green arrow indicating direct NO emission from soil to the soil-atmosphere interface has been added to the mechanism schematic (now Figure 3 in the revised manuscript). The figure legend now explicitly states as such.

<u>**Additional changes**</u>

Fig. 2C: On re-examination of the raw data for the 0 μM addition (control) samples, several of the $NO_2 + NO_z$ measurements fell below the detection limit of the Teledyne T200U NOy analyser. For these points we substituted the detection limit value (0.05 ppb) as the sample concentration when calculating the mean across the incubation and recalculated the corresponding flux accordingly. Figure 2C now reflects these revised control values, and the raw data are provided in the Fig2C tab of the source data file.

Superoxide analysis: The previous version of the manuscript incorrectly stated that the superoxide values were averaged across the hydroethidine and cytochrome c methods. However, the values used throughout are derived exclusively from the hydroethidine assay. The cytochrome c method was also performed, but a

number of its readings fell below the limit of detection, which would have introduced bias had the two methods been averaged. The trend is consistent between the two methods, as shown in the Fig1B tab of the supplementary data file. We have corrected the Methods to state explicitly that only hydroethidine-derived values were used.
